# Time Course of Improvement of Metabolic Parameters after a 12 Week Physical Exercise Programme in Patients with Type 2 Diabetes: The Influence of Gender in a Nigerian Population

**DOI:** 10.1155/2013/310574

**Published:** 2013-08-29

**Authors:** A. F. Adeniyi, A. E. Uloko, O. O. Ogwumike, A. O. Sanya, A. A. Fasanmade

**Affiliations:** ^1^Department of Physiotherapy, College of Medicine, University of Ibadan, Ibadan 200211, Nigeria; ^2^Department of Medicine, Aminu Kano Teaching Hospital, Kano 700233, Nigeria; ^3^Department of Medicine, College of Medicine, University of Ibadan, Ibadan 200211, Nigeria

## Abstract

Gender is a major determinant of the outcomes of many health interventions. This study documents the order of significant improvements in metabolic parameters of patients with type 2 diabetes mellitus (T2DM) having metabolic syndrome within 12 weeks of physical exercise programmes. Twenty-nine patients, mean age 49.6 ± 3.7 years, presenting with high fasting plasma glucose, high triglycerides, hypertension, and high waist circumference undertook a thrice weekly aerobic and endurance exercise programme in addition to their drugs and diet. Variables were assessed at baseline and end of every two weeks for twelve weeks. Compared with baseline, significant improvement (*P* < 0.05) in the metabolic parameters occurred in this order for the male participants: fasting glucose (2nd week), triglycerides and waist circumference (4th week), and systolic blood pressure (12th week). For the female participants, it was fasting glucose (4th week), triglycerides (6th week), and waist circumference (10th week). Regardless of the gender, fasting glucose was the first to improve significantly, followed by triglycerides. Hypertension did not improve significantly at all in the female participants as they may require more than twelve weeks of therapeutic exercise for any significant improvement in hypertension.

## 1. Introduction

In accordance with the operational definition provided by the National Cholesterol Education Program Adult Treatment Panel III (NCEP ATP III), an individual has the metabolic syndrome if three or more of high blood pressure, high blood glucose, high plasma triglycerides, low high density lipoprotein, high cholesterol, and high waist circumference are present [1–3]. Hence, metabolic syndrome is a clustering of obesity, diabetes, hyperlipidaemia, and hypertension [4]. The components of metabolic syndrome are quite interknitted, and the more of these conditions an individual has, the higher the risks of the individual for type 2 diabetes, heart diseases, and premature mortality [3, 5]. The disorder is of utmost public health importance because it is occurring in increasing frequency across the global population [4, 6]. The global increase in prevalence of the metabolic syndrome which is rampant in both industrialized and developing countries is also associated with an increase in obesity [4].

The primary intervention for metabolic syndrome includes moderate calorie restriction to achieve weight loss, change in dietary composition, and moderate physical activity [7]. However, the effort required for a busy person to keep up physical exercise routine may be too difficult to sustain [8]. Because lifestyle changes can be difficult to implement and maintain, drug treatment including statins, angiotensin-converting enzyme inhibitors, angiotensin-II receptor blockers, and oral antidiabetic agents can be considered [8–10]. The administration of drugs forms the secondary intervention, but specific drugs to take care of all the components at once are not available; hence, drugs to take care of each of the components are used [7]. 

Aerobic exercise training in patients with the metabolic syndrome can be useful as a treatment strategy and provides support for the role of physical activity in the prevention of chronic disease [5]. Consequently, exercise training should be considered an essential part of therapeutic lifestyle change and may concurrently improve insulin resistance and the entire cluster of metabolic risk factors [11]. Although some reports [4, 5, 9–13] among many others have alluded to the fact that physical exercise is beneficial for patients with the metabolic syndrome, most studies were not able to report the point within the exercise programme when each component of the metabolic syndrome shows the first significant improvement. This information is needed for making projections and monitoring the response of participants to interventions. This study was therefore conducted to determine the pattern of improvements and the durations within a 12-week physical exercise programme when each of the components of the metabolic syndrome will show the first significant improvement when compared with their baseline preexercise levels.

## 2. Materials and Methods

### 2.1. Participants

The participants who completed this study were 29 persons with type 2 diabetes mellitus (T2DM) attending the Specialty (Diabetic) Clinic of the Aminu Kano Teaching Hospital, (AKTH), Kano, Nigeria. They were made up of previously sedentary patients who were referred for the exercise programme after having met the preset eligibility criteria. Because all the participants were known patients with T2DM, it was considered that each prospective participant must have at least three other components of the metabolic syndrome from the ones specified by the NCEP ATP III Expert Panel on Detection, Evaluation, and Treatment of High Blood Cholesterol in Adults 2001 [2]. The three other components included raised triglyceride levels (over 1.7 mmol/L), high waist circumference (over 102 cm (men) or 88 cm (women)), and blood pressure (over 130/85 mmHg) [2]. Other eligibility criteria included being previously on oral medications and diet for the metabolic syndrome, not presenting with complications that will limit participation in the exercise programme such as blindness and amputation, willingness to take part in all the three sessions of the weekly exercise and fortnight assessment sessions, and not getting involved in any additional exercise programme aside the one offered during the study. Additionally, patients were expected to have been diagnosed as having T2DM for a minimum of 6 months and have a history of uncontrolled metabolic parameters with consistent medication type and dosage over a period of at least two weeks prior to the commencement of the study. Fifty-eight persons initially qualified for the study, and 32 participants commenced, but only 29 of them completed the study. The participants were recruited as they became available over a period of 18 months. Just before the exercise programme, each participant's cardiorespiratory fitness was determined by the fitness index using the Harvard step test [14]. The fitness index formula is duration of step bench exercise (in seconds) × 100/2 × the sum of any three pulse counts [14]. The higher the index, the better the person's fitness. Participant whose index was less than 65 was considered not physically fit and was excluded from the study [14].

### 2.2. Procedure for Data Collection

This study was an experimental study and it included the pretest, intervention and posttest phases. Ethical approval was sought and obtained for this study from the University of Ibadan/University College Hospital Research Ethics Committee (Protocol ID: UI/IRC/05/0105) and the Ethical Committee on Research of the Aminu Kano Teaching Hospital (Protocol ID: AKTH/MAC/SUB12/I/P.3). Consent of the physicians attending to the patients as well as the informed consent of the participants was also sought and obtained after due explanation of the nature, purpose, and procedure of the study. Purposive recruitment of participants for the study was done at the diabetes specialty clinic of Aminu Kano Teaching Hospital, Kano, while all the exercise sessions were carried out at the gymnasium of the outpatient Physiotherapy Clinic of the same hospital. Laboratory analyses were carried out at the Chemical Pathology Laboratory of the same hospital.

### 2.3. The Data Collection

Demographic and lifestyle data of the participants including age, sex, smoking habit, and duration of diagnosis of T2DM were recorded. Baseline data on all the selected clinical variables were also taken at baseline before the commencement of the exercise and subsequently at the end of the 2nd, 4th, 6th, 8th, 10th, and 12th weeks of the exercise programme. The clinical variables that were assessed at baseline and in the subsequent assessment periods include those that were assessed from the gymnasium and those that were analysed in the chemical pathology laboratory of the hospital. The data that were assessed in the gymnasium include the body mass index, waist circumference, waist hip ratio, body fat percentage, and systolic and diastolic blood pressures. The data that were analysed in the laboratory include the fasting plasma glucose, glycosylated haemoglobin (HBA1_c_), high and low density lipoproteins, triglycerides, and total cholesterol. The body mass index, waist circumference, waist hip ratio, body fat percentage, and systolic and diastolic blood pressures were all assessed using previously documented procedures [15–18]. The laboratory analyses of fasting plasma glucose, HBA1_c_, and lipid profile analyses were all carried out using standard procedures. 

### 2.4. The Exercise Programme

The participants received a regimen of therapeutic exercise in addition to their prescribed drugs and diet. The total duration of exercise per session was about 45 minutes, and each participant went through the sessions for three days in a week for 12 weeks. Exercises were undertaken on alternate days, and patients were encouraged not to allow more than a day interval between sessions (except weekends). Each session of the exercise was made up of additional ten minutes of warm-up and cooldown exercises of five minutes each. The warm-up phase included slow-paced walking around the gymnasium, side bends, and waist twists, while cool-down included breathing exercises, spine and trunk twists, and gentle walking [19, 20]. The exercise programme included the following.

#### 2.4.1. Aerobic Exercise I

The participants pedalled a bicycle ergometer beginning with an intensity of 60% of heart rate reserve; that is, [0.6 × (Heart Rate_max_ − Heart Rate_rest_) + Heart Rate_rest_] for 20 minutes [21]. This was broken into two parts of 10 minutes each with two minutes break [20]. This intensity of exercise was progressed gradually based on the participant's self-selected capacity to progress.

#### 2.4.2. Aerobic Exercise II

This included two rounds of brisk walking with full arm swings up and down the length of the gymnasium (about ten meters long) [22] at the patients self-selected speed. Minimum of three minutes rest was observed between the two laps by the participants.

#### 2.4.3. Mobilization Exercise

Free active mobilization of the joints of the shoulder, elbow, wrists, fingers, hip, knees, and ankle was carried out to as full range as possible [23, 24]. Participants were encouraged by the researchers to mobilize the joints in the movement planes as full as possible making ten repetitions in each.

#### 2.4.4. Resistance Exercise

Strengthening exercises of the muscles of the upper limbs and lower limbs were carried out in this session. 60% of the individual's one repetition maximum (1 RM) was determined and used to strengthen each group of muscles. Participants were asked to perform two sets of ten repetitions each with recovery time of two minutes between sets [25, 26]. The resistance regimen was done for the flexors and extensors of the knee and elbows [26–28]. 

#### 2.4.5. Progression of the Exercise Programmes and Medications

Upward review of the initial intensity of 60% of heart rate reserve was made to 65% and 70% at the beginning of the fifth and ninth weeks, respectively, [21] for the bicycle ergometry. New 1 RM was determined every fortnight, and 60% of that was utilized thereafter for the strengthening exercise [28]. In the course of the 12 week exercise programme, the physicians looking after the patients were allowed to review the patients and adjust their medications as necessary. 

### 2.5. Analysis of Data

Categorical data of the participants were presented in frequencies and percentages, while quantitative values for each of the demographic and clinical variables were presented in the form of mean and standard deviations, minimum and maximum values, and confidence intervals. In addition to the baseline assessment, the selected metabolic parameters were reassessed every two weeks for 12 weeks in both the male and female groups. The patterns of changes in the mean values of the metabolic parameters from the baseline to the 12th week are presented using line charts with standard errors. The parameter readings for the male and female groups at every two-week interval were each compared with the original baseline readings for each group using the paired *t*-test. The point at which the first significant improvement occurred for each parameter was noted for the male and female groups. Data were analysed using the Statistical Package for the Social Sciences (SPSS) Version 15 (SPSS Inc, Chicago, IL, USA.). Level of significance was set at *P* < 0.05. 

## 3. Results

### 3.1. Characteristics of the Participants

In the entire duration of the study spanning 18 months, 58 persons initially qualified for the study based on the eligibility criteria, and they also agreed to participate. However, 26 (44.8%) of the number declined participation when the details of the exercise programme were explained to them. A total of 29 participants completed the exercise programme representing 90.6% of the 32 participants who started it. The mean ages of the male and female participants were 46.2 ± 3.7 and 51.5 ± 6.4 years, respectively, (Table 1), and 18 (62.1%) of the participants were aged 40 years and above. Most (72.4%) of the participants were females, and the mean age of diagnosis for the female participants was 7.7 ± 2.0 years with 34.5% of the participants being diagnosed with T2DM for a minimum of 5 years. None of the female participants were smokers, but 3 (37.5%) out of the 8 male participants were smokers of tobacco. A highlight of the medications received by the participants at the beginning of the study is also presented in Table 1. The list of medications covers the ones administered for diabetes, dyslipidaemia, and hypertension. The table also contains the summary of the known complications presented by the participants. 

### 3.2. Data on the Clinical Variables of the Participants at Baseline

Before the commencement of the exercise programme, each participant's clinical data were taken and presented by gender (Table 2). The fasting plasma glucose in women (10.6 ± 3.9 mmol/L) was higher than that of the male participants (8.9 ± 2.1 mmol/L). A similar trend is also seen for the HBA1_c_ readings where that of the female participants was higher than that of the male participants. At baseline, the mean body mass index of the male (29.6 ± 5.4 kg/m^2^) and female (35.8 ± 6.9 kg/m^2^) participants placed them as overweight and obese, respectively. Similarly, the mean of waist circumference for both the male (103 ± 11.1 cm) and female (96.5 ± 8.6 cm) participants placed both groups as having substantially increased risk for cardiometabolic disorders. Details of other clinical variables comprising waist hip ratio, body fat percentage, high and low density lipoprotein, total cholesterol, triglycerides, and blood pressure are presented in Table 2. 

The fitness index of the participants increased significantly (*P* < 0.05) from 67.8 ± 7.4 at baseline to 81 ± 3.77 at the end of 12 weeks. 1 RM of the flexors and extensors of the knees and elbows at the end of 12 weeks also increased significantly (*P* < 0.05) when compared with the baseline values. At the end of the 12 weeks of exercise programme, compared with the baseline, the mean weight of both the male and female participants also changed significantly (*P* < 0.05) from 83.9 ± 11.2 kg to 71.6 ± 8.0 kg and from 91.5 ± 9 kg to 84.6 ± 4.4 kg, respectively.

### 3.3. Changes in the Metabolic Parameters of Participants across the 12 Weeks of Exercise Programme

**Figure 1:**
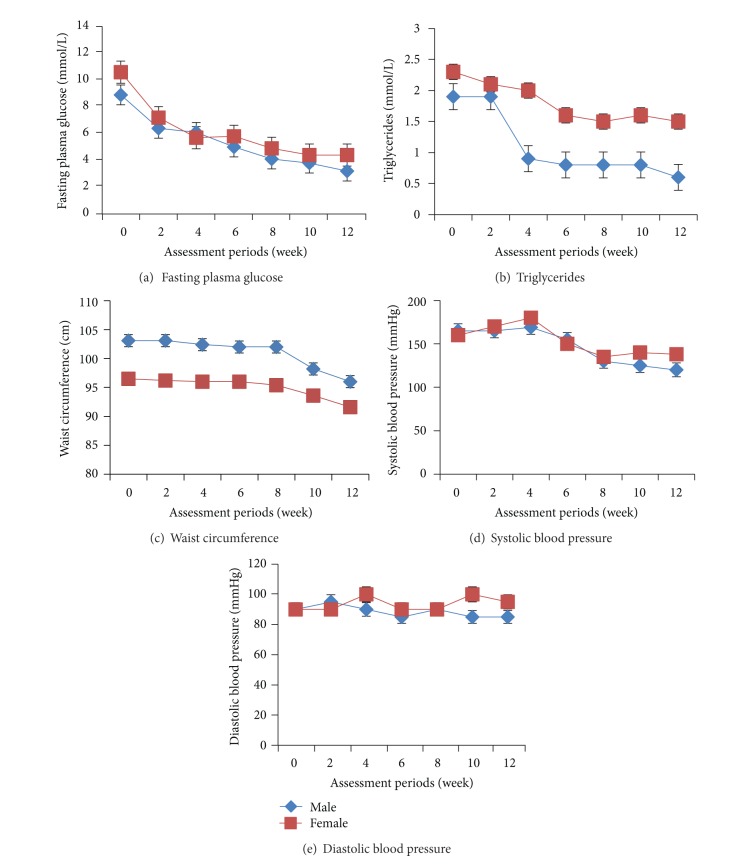
Changes in the mean values of the metabolic parameters by gender across the 12 weeks of exercise programme (all shown with standard errors).

The four selected components of the metabolic syndrome of interest in this study include fasting plasma glucose, waist circumference, triglycerides, and blood pressure. In addition to the baseline assessment, the variables were also reassessed at two week intervals from the 2nd week to the 12th week.  Figure 1(a) shows the changes in fasting plasma glucose from baseline to the end of the 12th week of exercise programme. The figure shows a drop in the fasting plasma glucose of both male (from 8.9 to 6.4 mmol/L) and female (from 10.6 to 7.2 mmol/L) participants by the 2nd week, and this continued steadily to the end of the 12th week. The situation is a bit different in the observation of the triglycerides (Figure 1(b)). The male participants initially did not respond in the first two weeks compared to the female participants which witnessed a drop in triglycerides from 2.3 to 2.1 mmol/L at the 2nd week. However, by the 4th week, there was a steep drop in triglycerides among the male participants (from 1.9 to 0.9 mmol/L), and this surpassed that of the female participants. Figures 1(c), 1(d), and 1(e) also present the patterns for the waist circumference, systolic blood pressure, and diastolic blood pressure, respectively. The pattern of changes in the diastolic blood pressure appears to be the most inconsistent of all the patterns presented.

Within each gender group, the metabolic parameter readings at every two weeks interval were each recompared with the baseline using the paired *t*-test to detect the point when the first significant (*P* < 0.05) improvement occurred (Table 3). The first significant improvement was observed in the fasting plasma glucose of both male (*t* = 8.059; *P* = 0.0001) and female groups (*t* = 13.007; *P* = 0.01) by the 2nd and 4th weeks, respectively. While the triglyceride and waist circumference significantly improved simultaneously at the 4th week for the male participants, the female participants had their first significant improvement of triglyceride (*t* = 5.549; *P* = 0.002) at the 6th week and waist circumference (*t* = 5.492; *P* = 0.001) at the 10th week. The systolic was the only blood pressure that improved significantly among the male participants, and it was observed at the 12th week. The female participants never had any significant improvement in blood pressure throughout the 12 weeks.

## 4. Discussion

This study was conducted to determine the pattern of changes in selected components of the metabolic syndrome among patients with T2DM over a period of 12 weeks of physical exercise programme and to determine the relative duration of first significant improvement in each of the components based on gender considerations. The findings from the study indicate that (1) most of the clinical parameters including those that are not metabolic parameters demonstrated either clinical or significant improvement over the 12 week exercise programme, (2) fasting plasma glucose was the first to show significant improvement in both the male (second week) and female (fourth week) participants, and (3) systolic was the last and only blood pressure to show significant improvement (twelfth week) and only among the male participants. 

The values of the glycaemic, adiposity, and lipid profile parameters of the T2DM patients in this study were on the high side at baseline compared with what is generally documented as normal values [1–3], while the high density lipoprotein was lower than the required minimum. From Nigeria, where this study was conducted, the Federal Ministry of Health [29] published the Standard Treatment Guideline which designates a fasting plasma level of ≥7 mmol/L as signifying diabetes mellitus, and the mean of fasting blood glucose of the participants at baseline was higher than this. One of the possible reasons for the inappropriate levels of the parameters could be attributed to the report of the NCEP ATP III which considered the “obesity epidemic” as mainly responsible for the rising prevalence of metabolic syndrome [30]. The report claims that obesity contributes to hypertension, high serum cholesterol, low high density lipoprotein, and hyperglycaemia, and it is otherwise associated with higher cardiovascular disease risk. In agreement with this assertion, it was observed that the mean body mass index of participants in this study actually classified the men as overweight and the women as obese. 

All the parameters that were considered in this study demonstrated improvement over the 12-week exercise period. The pattern of improvement over the 12-week period was however varied. While some of the parameters such as fasting plasma glucose and waist circumference improved consistently over the 12 week period, some other parameters like triglycerides, systolic and diastolic blood pressures were seen to be inconsistent with periods of improvement seen to be interposed with periods of relapses. In a meta-analysis, lifestyle modification intervention including exercise significantly reduced mean values for waist circumference by −2.7 cm, fasting blood glucose by −11.5 mg/dL, systolic blood pressure by −6.4 mmHg, diastolic blood pressure by −3.3 mmHg, and triglycerides by −12.0 mg/dL [6]. 

Of all the metabolic parameters, fasting glucose was the first to show significant improvement among both the male and female participants. The reason why fasting plasma glucose was the first to undergo significant improvement is not immediately known, but the fact that the exercise programme in this study combined two important exercise programmes may have played a significant role. Exercise training has been shown to favourably affect glycaemic parameters [31, 32], and both aerobic and resistance exercise protocols have been reported to be effective in reducing pre- and postexercise blood glucose and HBA1_c_ levels, with resistance exercise producing more significant reductions [33]. A previous study also spanning 12 weeks of exercise programme had reported that blood glucose control and insulin sensitivity did not change during the study period [34]. It was however observed that the exercise programme of Ligtenberg et al. [34] did not include a resistance programme. In a study that combined aerobic and resistance exercise like the present study, significant reductions were equally observed in glucose after four weeks of training [35]. That blood glucose was the first to show significant drop in this present study may also be because of the high baseline glucose level. A previous study had linked a decrease in blood glucose within a four week aerobic and resistance exercise programme to a baseline blood glucose level greater than 8.85 mmol/L [36]. 

Triglyceride and waist circumference simultaneously demonstrated their first significant improvement in the 4th week among the male participants and this improvement occurred faster compared to the female participants. While it was observed in this study that the male and female participants had significant improvement in their triglycerides in the 4th and 6th week, respectively, Ligtenberg et al. [34] reported though not by gender that levels of total triacylglycerols, very-low-density lipoprotein-triacylglycerols, and apolipoprotein B were significantly lower after 6 weeks. A somewhat similar experience in gender variation was also reported when triglyceride concentrations were 30.92 mg/dL lower in men (*P* = 0.029) and 12.83 mg/dL lower in women (*P* = 0.003) who had high physical activity levels when compared with less active individuals [37]. Lahti-Koski et al. [38] reported that waist circumference increased by 2.7 cm in men and 4.3 cm in women over a 15-year period and women have been reported as having substantially more total adipose tissue than men [39]. Based on these observations, it may actually take a longer time to have reductions of triglycerides and waist circumferences among female compared to male participants.

Blood pressure appeared to be the most intractable of the metabolic syndrome components considered in this study, because only the systolic pressure of only the male participants improved significantly, and this occurred only at the 12th week. This observation has failed to corroborate earlier reports where the decline in blood pressure was significant statistically and clinically after walking [40] and treadmill [41] exercise programmes that were similarly conducted over a similar duration of 12 weeks. The entire reasons why blood pressure appeared recalcitrant in this study is not known, but some reasons will be provided. The significant improvement seen in the studies by Hua et al. [40] and Dimeo et al. [41] may well be because the patients were not having comorbidities to the hypertension unlike the patients in this present study where the individuals had a number of other comorbidities. A similar explanation had been offered where diabetes and chronic renal disease in combination with other possible extrinsic and intrinsic factors including abnormal neurohormonal regulation, lack of physical activity, increased dietary sodium intake, and smoking of tobacco have been implicated for lack of improvement in blood pressure [30, 42].

The findings from this study have a number of clinical applications for T2DM patients who have the metabolic syndrome. The first clinical application of this study is its possible use for prognostic purposes among male and female patients who are placed on aerobic and resistance exercise programmes in addition to their routine medications and diet. The various durations when the first significant improvements were noticed for each of the variables could serve as a possible guide for making projections into the minimum duration when significant improvements are expected to occur for each of the metabolic parameters. The second potential clinical application is its ability to be used for monitoring the effectiveness of similar treatment packages. Inability to see significant improvements as at the durations specified in this study may therefore require further scrutiny of the patient. Thirdly, the outcomes of this study may be used to allay the fear of anxious patients who will express concerns when improvement in some metabolic parameters is not significant in line with their own projected expectations. This study further confirms that not only did the exercise programmes affect acute changes in blood lipids, blood pressure, and glucose homeostasis [32], but they also exhibited chronic adaptations on the parameters, and this corroborates some previous studies [34, 35]. 

This study has some limitations which should be put into perspective in order for the findings to be applied judiciously. It is imperative to state that this study was conducted only within a 12-week exercise programme. Hence, the pattern of recovery if the exercise is extended beyond 12 weeks is not evidently known. It is however expected that the improvements will be sustained as long as the exercise programme is sustained. It had been reported previously that adaptations from exercise were further improved after 16 weeks for both glucose and glycated haemoglobin [35]. It is also important to note that this study could not feature a control group as well as a equal number of male and female participants in each of the groups. These were borne out of the strict eligibility criteria which adversely affected the number of patients that were qualified for this study. Nevertheless, it should be noted that this study was not conducted to investigate the effectiveness of exercise in metabolic syndrome as this has already been presented generously in various literature [4, 5, 9–13]. All that was investigated was the order of the improvements of the metabolic parameters, and identifying the order was possible with the design of this study. It is also important to note that the participants were taking their medications during the exercise programme. This implies that the observed durations of first improvement of the metabolic parameters should only be expected to reoccur if the patient is on similar therapy programmes. The lack of a “no medication” control group makes it difficult to ascribe the improvement in metabolic parameters to exercise alone as changes may have been influenced by their medications. It was however observed that all the patients witnessed a decrease in dosage and/or combinations of their medications throughout the study period. The lack of a “no medication” control group for the study was basically an ethical issue as it was difficult asking patients to stop medications during the study. In addition, the point of significant improvement in the parameters was taken as the point when the parameters witnessed the first significant improvement when compared with the baseline and not necessarily when the parameter dropped to the cutoff value, and it was based on the male and female group considerations and not on individual basis. 

It is concluded that both the male and female patients with T2DM who went through the 12 week aerobic and resistance exercise programme experienced significant improvements in the components of the metabolic syndrome at different stages within the 12 weeks. Compared to the male participants, the first significant improvements among the female participants were delayed, and the female participants did not experience any significant improvement in their blood pressure.

## Figures and Tables

**Table 1:** Descriptive characteristics including medications of the participants by gender.

Variable	Mean ± SD	Minimum	Maximum	95% CI
Age (years)				
Male (*n* = 8)	46.2 ± 3.7	32.0	56.0	39.0–47.0
Female (*n* = 21)	51.5 ± 6.4	28.0	67.0	41.0–55.0
Length of diagnosis of diabetes (years)				
Male (*n* = 8)	4.9 ± 1.6	0.5	8.0	2.0–5.0
Female (*n* = 21)	7.7 ± 2.0	0.8	11.0	1.2–7.8

	*Medications			
	Number of medications at baseline	Medications at baseline		

Diabetes medications				
3 males, 5 Females	3	Metformin, glimepiride, insulin		
5 males, 11 Females	2	Metformin, glimepiride,		
5 Females	3	Metformin, glyburide, insulin		
Lipid medications				
14 females	2	Atorvastatin, fenofibrate		
5 males, 4 females	1	Atorvastatin		
3 males, 3 females	1	Fenofibrate		
Blood pressure medications				
6 males, 9 females	2	Amlodipine, valsartan		
2 males, 5 females	2	Lisinopril, bendrofluazide		
7 females	2	Hydrochlorothiazide losartan		

	Known complications			
	Number of complications	Types of complications		

3 males, 5 females	3	Nephropathy, PN, PAD		
4 males, 12 females	1	PN		
4 females	4	IHD, PN, nephropathy, PAD		

Key: IHD: ischaemic heart disease; PN: peripheral neuropathy; PAD: peripheral arterial disease.

*Types of medications were in various combinations based on the physician's assessment of the need of individual patient and in response to the exercise programme; downward adjustments were made in combinations or dosages as deemed necessary by the physician. There was no upward review of dosage or combinations throughout the study period.

**Table 2:** Clinical profile of the participants at baseline by gender.

Variable	Mean ± SD	Minimum	Maximum	95% CI
Fasting plasma glucose (mmol/L)				
Male (*n* = 8)	8.9 ± 2.1	2.0	18.0	6.6–8.0
Female (*n* = 21)	10.6 ± 3.9	3.0	22.0	9.4–11.9
HBA1_c_ (%)				
Male (*n* = 8)	8.79 ± 2.69	6.38	11.06	8.22–9.14
Female (*n* = 21)	10.08 ± 1.72	7.20	13.60	9.55–10.61
Body mass index (Kg/m^2^)				
Male (*n* = 8)	29.6 ± 5.4	23.5	52.4	29.3–38.8
Female (*n* = 21)	35.8 ± 6.9	26.1	57.3	31.5–39.9
Waist circumference (cm)				
Male (*n* = 8)	103.1 ± 11.1	75.0	141.0	98.5–106.8
Female (*n* = 21)	96.5 ± 8.6	76.0	101.0	89.2–98.6
Waist hip ratio				
Male (*n* = 8)	0.95 ± 0.05	0.88	1.03	0.95–0.97
Female (*n* = 21)	0.91 ± 0.02	0.83	0.95	0.85–0.89
Percent body fat (%)				
Male (*n* = 8)	33.24 ± 5.28	22.30	42.70	30.87–36.33
Female (*n* = 21)	37.91 ± 4.78	18.70	47.80	26.16–40.94
High density lipoprotein (mmol/L)				
Male (*n* = 8)	0.76 ± 0.23	0.65	1.60	0.62–0.86
Female (*n* = 21)	0.71 ± 0.35	0.58	1.86	0.66–0.84
Low density lipoprotein (mmol/L)				
Male (*n* = 8)	3.92 ± 0.79	1.30	5.60	3.53–4.02
Female (*n* = 21)	4.35 ± 1.10	1.58	6.37	2.74–4.82
Total cholesterol (mmol/L)				
Male (*n* = 8)	7.17 ± 1.52	2.90	9.60	6.70–7.64
Female (*n* = 21)	8.22 ± 1.97	1.52	9.86	4.18–6.72
Triglyceride (mmol/L)				
Male (*n* = 8)	1.9 ± 0.3	1.0	2.3	1.6–2.1
Female (*n* = 21)	2.3 ± 0.5	1.2	3.1	1.8–2.6
Systolic blood pressure (mmHg)				
Male (*n* = 8)	165.0 ± 7.50	140.00	190.00	156.5–170.5
Female (*n* = 21)	160.0 ± 6.00	120.00	210.00	140.0–180.0
Diastolic blood pressure (mmHg)				
Male (*n* = 8)	90.00 ± 8.30	75.00	110.00	80.0–95.0
Female (*n* = 21)	90.00 ± 10.5	80.00	100.00	90.0–95.0

**Table 3:** First significant changes in metabolic parameters by gender (reassessment values compared with baseline).

	Second week	Forth week	Sixth week	Eighth week	Tenth week	Twelfth week
Sex						
Male (*n* = 8)	Fasting glucose *t* = 8.059 *P* = 0.0001	Triglycerides *t* = 8.327 *P* = 0.002 Waist circumference *t* = 13.869 *P* = 0.01				Systolic blood pressure *t* = 5.874 *P* = 0.03
Female (*n* = 21)		Fasting glucose *t* = 13.007 *P* = 0.01	Triglycerides *t* = 5.549 *P* = 0.002		Waist circumference *t* = 5.492 *P* = 0.001	

## References

[1] Cleeman JI (2001). Executive summary of the third report of the National Cholesterol Education Program (NCEP) expert panel on detection, evaluation, and treatment of high blood cholesterol in adults (adult treatment panel III). *Journal of the American Medical Association*.

[2] (2002). Third report of the National Cholesterol Education Program (NCEP) expert panel on detection, evaluation, and treatment of high blood cholesterol in adults (Adult Treatment Panel III). Final report. *Circulation*.

[3] Canadian Diabetes Association Metabolic syndrome. http://www.diabetes.ca/diabetes-and-you/what/metabolic-syndrome/.

[4] Golbidi S, Mesdaghinia A, Laher I (2012). Exercise in the metabolic syndrome. *Oxidative Medicine and Cellular Longevity*.

[5] Katzmarzyk PT, Leon AS, Wilmore JH (2003). Targeting the metabolic syndrome with exercise: evidence from the HERITAGE family study. *Medicine and Science in Sports and Exercise*.

[6] Yamaoka K, Tango T (2012). Effects of lifestyle modification on metabolic syndrome: a systematic review and meta-analysis. *BMC Medicine*.

[7] The IDF consensus worldwide definition of the metabolic syndrome. http://www.idf.org/webdata/docs/IDF_Meta_def_final.pdf.

[8] Opie LH (2007). Metabolic syndrome. *Circulation*.

[9] Haffner S (2006). Diabetes and the metabolic syndrome—when is it best to intervene to prevent?. *Atherosclerosis Supplements*.

[10] Pitsavos C, Panagiotakos D, Weinem M, Stefanadis C (2006). Diet, exercise and the metabolic syndrome. *Review of Diabetic Studies*.

[11] Carroll S, Dudfield M (2004). What is the relationship between exercise and metabolic abnormalities? A review of the metabolic syndrome. *Sports Medicine*.

[12] Thomas TR, Warner SO, Dellsperger KC (2010). Exercise and the metabolic syndrome with weight regain. *Journal of Applied Physiology*.

[13] Touati S, Meziri F, Devaux S, Berthelot A, Touyz RM, Laurant P (2011). Exercise reverses metabolic syndrome in high-fat diet-induced obese rats. *Medicine and Science in Sports and Exercise*.

[14] Magee DJ (1992). *Orthopaedics Physical Assessment*.

[15] Hall JG, Froster-Iskenius VG, Allanson JE (1989). *Handbook of Normal Physiological Measurements*.

[16] Martín MV, Gómez GB, Antoranz GM, Fernández HS, Gómez DL, de Oya OM (2001). Validation of the OMRON BF 300 monitor for measuring body fat by bioelectric impedance. *Atencion Primaria*.

[17] Manual O (2004). *Body Fat Monitor Instruction Manual*.

[18] Wilmore JH, Costill DL *Physiology of Sport and Exercise*.

[19] Davis D, Kimmet T, Auty M (1989). *Physical Education: Theory and Practice*.

[20] Cuff DJ, Meneilly GS, Martin A, Ignaszewski A, Tildesley HD, Frohlich JJ (2003). Effective exercise modality to reduce insulin resistance in women with type II diabetes. *Diabetes Care*.

[21] Wilson PK, Fardy PS, Froelider VF (1981). *Cardiac Rehabilitation, Adult Fitness and Exercise Testing*.

[22] Sigal RJ, Kenny GP, Wasserman DH, Castaneda-Sceppa C (2004). Physical activity/exercise and type 2 diabetes. *Diabetes Care*.

[23] Sanya AO (1998). The role of physiotherapy in the total management of diabetes mellitus. *Journal of Nigeria Medical Rehabilitation Therapists*.

[24] Goldsmith JR, Lidtke RH, Shott S (2002). The effects of range-of-motion therapy on the plantar pressures of patients with diabetes mellitus. *Journal of the American Podiatric Medical Association*.

[25] Dunstan DW, Daly RM, Owen N, Jolley D, De Courten M, Shaw J (2002). High-intensity resistance training improves glycemic control in older patients with type 2 diabetes. *Diabetes Care*.

[26] Brandon LJ, Gaasch DA, Boyette LW, Lloyd AM (2003). Effects of long-term resistive training on mobility and strength in older adults with diabetes. *Journals of Gerontology A*.

[27] Hazeldine R (1990). *Strength Training for Sport*.

[28] Lycholat T (1990). *The Complete Book of Resistance Training*.

[29] Standard treatment guidelines.

[30] Grundy SM, Brewer HB, Cleeman JI, Smith SC, Lenfant C (2004). Definition of Metabolic Syndrome: report of the National Heart, Lung, and Blood Institute/American Heart Association Conference on Scientific Issues Related to Definition. *Circulation*.

[31] Jorge MLMP, De Oliveira VN, Resende NM (2011). The effects of aerobic, resistance, and combined exercise on metabolic control, inflammatory markers, adipocytokines, and muscle insulin signaling in patients with type 2 diabetes mellitus. *Metabolism*.

[32] Thompson PD, Crouse SF, Goodpaster B, Kelley D, Moyna N, Pescatello L (2001). The acute versus the chronic response to exercise. *Medicine and Science in Sports and Exercise*.

[33] Bweir S, Al-Jarrah M, Almalty A (2009). Resistance exercise training lowers HbA1c more than aerobic training in adults with type 2 diabetes. *Diabetology and Metabolic Syndrome*.

[34] Ligtenberg PC, Hoekstra JBL, Bol E, Zonderland ML, Erkelens DW (1997). Effects of physical training on metabolic control in elderly type 2 diabetes mellitus patients. *Clinical Science*.

[35] Tokmakidis SP, Zois CE, Volaklis KA, Kotsa K, Touvra A-M (2004). The effects of a combined strength and aerobic exercise program on glucose control and insulin action in women with type 2 diabetes. *European Journal of Applied Physiology*.

[36] Hordern MD, Cooney LM, Beller EM, Prins JB, Marwick TH, Coombes JS (2008). Determinants of changes in blood glucose response to short-term exercise training in patients with type 2 diabetes. *Clinical Science*.

[37] Dancy C, Lohsoonthorn V, Williams MA (2008). Risk of dyslipidemia in relation to level of physical activity among Thai professional and office workers. *Asian Journal of Tropical Medicine and Public Health*.

[38] Lahti-Koski M, Harald K, Männistö S, Laatikainen T, Jousilahti P (2007). Fifteen-year changes in body mass index and waist circumference in Finnish adults. *European Journal of Cardiovascular Prevention and Rehabilitation*.

[39] World Health Organization (WHO) Waist circumference and waist hip ratio: report of a WHO expert consultation. http://whqlibdoc.who.int/publications/2011/9789241501491_eng.pdf.

[40] Hua LPT, Brown CA, Hains SJM, Godwin M, Parlow JL (2009). Effects of low-intensity exercise conditioning on blood pressure, heart rate, and autonomic modulation of heart rate in men and women with hypertension. *Biological Research for Nursing*.

[41] Dimeo F, Pagonas N, Seibert F, Arndt R, Zidek W, Westhoff TH (2012). Aerobic exercise reduces blood pressure in resistant hypertension. *Hypertension*.

[42] Kanbay M, Turgut F, Erkmen Uyar ME, Akcay A, Covic A (2008). Causes and mechanisms of nondipping hypertension. *Clinical and Experimental Hypertension*.

